# Psychological Symptoms in Family of Death Patients with infectious diseases

**DOI:** 10.1192/j.eurpsy.2022.2280

**Published:** 2022-09-01

**Authors:** G. Lacatusu, C. Sapaniuc, D. Manciuc

**Affiliations:** 1 Hospital of Infectious Diseases “Sf. Parascheva”, Infectious Diseases, Iasi, Romania; 2 Universiity of Medicine and Pharmacy “Gr. T. Popa”, Infectious Diseases, iasi, Romania

## Abstract

**Introduction:**

Looking back into history, infectious diseases played an important role in human history being responsible, in terms of pathologies, for more deaths than any other disease.

**Objectives:**

Considering that infectious diseases have a high rate of transmissibility, with an acute debut and sometimes with a fast evolution to exitus, the impact of the news on families of the departed patient diagnosed with an infectious disease can come as a shock. We conducted a literature review regarding the the psychological symptoms, moruning and staging of processing a loss using the international database.

**Methods:**

We conducted a literature review regarding the the psychological symptoms, moruning and staging of processing a loss using the international database.

**Results:**

Processing the unexpected death of a family member needs not only the implication of the physician but also the counseling of a specialized psychologist which can help the families through all stages of loss and grief.

**Conclusions:**

For the family of deceased patients in hospitals, mourning and depression are a reality that the psychologist and the attending physician face every day. The team of psychologists and medical doctors are facing cases of severe shock and depression in parents, varying with the age of the child and of the young adult, in cases with an acute or severe disease leading to death.

**Disclosure:**

No significant relationships.

